# Healthcare Students’ Exposure and Knowledge of the 4Ms Framework for an Age-Friendly Health System

**DOI:** 10.1093/geroni/igaf122.3953

**Published:** 2025-12-31

**Authors:** Laura Finn, Isabel Coulter-Daly, Leah Peterman

**Affiliations:** Philadelphia College of Pharmacy / St Joseph’s University, Philadelphia, Pennsylvania, United States; Philadelphia College of Pharmacy, LANGHORNE, Pennsylvania, United States; Philadelphia College of Pharmacy at Saint Josephs University, Philadelphia, Pennsylvania, United States

## Abstract

Pharmacists are essential healthcare professionals directly involved in the care of older individuals. To effectively and holistically fulfill the needs of these patients, pharmacists must possess a strong knowledge of the foundations of geriatric care; this is represented by the 4Ms Framework for an Age-Friendly Health System. The framework is one that all professionals should be exposed to, particularly those in the early stages of their careers as to adopt this approach for their future practice. The aim of this study at St. Joseph’s University was to evaluate pharmacy and physical therapy students’ familiarity with the 4Ms framework through an 18-question online survey that included multiple choice and open-ended responses. The results demonstrated that 88% percent of the surveyed healthcare students either never heard of the 4Ms framework or had heard of the approach but were unclear of its meaning. Findings highlight the lack of awareness future professionals have on how to properly care for an older individual. Almost 70% of responding students reported that their program either did not have a required geriatrics course or they were unsure if it did, and only 4% of participants took a geriatrics elective. These results emphasize that training and exposure to caring for this patient population should be incorporated into curricula, experiential programs, and extracurricular activities. Doing so will be beneficial to both students by increasing their preparedness and to older adult patients by improving the quality of their care.

